# The Level of Depression and Assertiveness among Nursing Students

**Published:** 2014-07

**Authors:** Fatemeh Rezayat, Nahid Dehghan Nayeri

**Affiliations:** 1 Department of Psychiatric Nursing, Tehran University of Medical Sciences, Tehran, Iran;; 2 Nursing and Midwifery Care Research Center, School of Nursing and Midwifery, Tehran University of Medical Sciences, Tehran, Iran

**Keywords:** Depression, Assertiveness, Nursing Student

## Abstract

**Background:** Nursing students are susceptible to a higher risk of depression. Recognition of depression, assertiveness and the relationship between them is important since if a relationship exists, probably enhancing the level of assertiveness can reduce depression in this high risk group. We aimed to investigate depression and assertiveness levels and the relationship between them in nursing students of Tehran University of Medical Sciences.

**Methods:** The target population of this cross-sectional study was bachelor nursing students of Tehran University of Medical Sciences, as the largest and one of the most prestigious Iranian universities. For selecting samples, the convenience sampling method was used. Having no previous information about classes, the researcher selected the students from the courses held while the researcher was present for sampling at the faculty and studied all the students willing to participate in the study in selected classes. The questionnaire consisted of demographic information, Gambrill and Richey assertion inventory, and Beck’s depression inventory. SPSS software, version 16, was used to analyze the data. ANOVA and independent samples *t* test were used for as appropriated.

**Results:** 55.6% of students indicated average and low levels of assertiveness and 38.7% were suffering from mild to severe depression. Pearson correlation test showed a significant inverse relationship (r=0.314 and P<0.001) between assertiveness and depression. There were significant relationships between depression and interest in the field of study (P=0.001) and between assertiveness and gender (P=0.035).

**Conclusion:** There was an inverse significant relationship between depression and assertiveness among nursing students. We suggest a cohort study design that can determine the effect of these variables on each other completely.

## Introduction

Stress, low assertiveness, depression, hostility, and aggressiveness are among the problems prevalent among young university students.^1^ Depression is a serious health issue and a considerable reason for restlessness among students. It affects their lives during the critical learning and social development process.^2^ Students are considered as a vulnerable group for the development of depression and its symptoms.^3^ This group deals with numerous types of stressors such as being separated from the family for the first time, residing in dormitory beside other students, experiencing freedom from the parents’ surveillance,^2^ obligation to succeed, as well as the anxiety over the future and problems of joining a social system. They also suffer from physical, emotional, familial, and social problems which can affect their learning process and academic performance and, as a result, increase their depression.^4^ Depression in the studentship period, which can negatively affect the professional future and social communications of the students, is closely related to their unstable communications, suicidal thoughts and attempts, and poor working performance.^5^ These factors, in turn, generate stress and exacerbate depression in students.^6^

The educational environment of medical sciences is recognized as stressful and usually has negative impacts on educational performance and feeling of well-being in students. There is a growing anxiety toward the emotional distress in medical students. Depression is a common experience among students, since one out of seven students suffers from depression.^7^ During the education period, medical students not only have the common problems of other students, but also deal with particular problems of their field of study including high mental and emotional pressures of the hospital and emergency unit, confronting with patients’ problems, and their lengthy academic period. Therefore, they are exposed to higher risk of mental health problems as compared with other students.^8^ In one study, researchers reported a prevalence of 60% and 49% for depression in nursing and medical students, respectively.^9^ In another study, depression was related to the individuals’ assertiveness.^10^

Assertiveness is among the treatable interpersonal communication aspects. This skill could empower individuals to efficiently communicate with their superior and subordinate colleagues.^11^ Assertiveness is defined as a verbal and nonverbal behavior enabling the individuals to follow their interests, stand on their own feet, and express their comments, feelings, and way of thought frankly without any stress. Assertiveness also includes respecting others’ rights.^12^ Assertive people respect and honor others; they are not passive and do not allow other people to manipulate them. Moreover, they respect others’ needs and demands and communicate with them tactfully.^13^ Lazarus believes that assertiveness consists of four components: demand rejection, seeking for others’ care and addressing their needs, positive and negative feelings, and the ability of commencing, resuming, and closing the conversions.^14^

Assertiveness is among the most important and essential social skills making a part of the extensive concept of interpersonal and behavioral skills. The simultaneous presence of low assertiveness and high anxiety in the students results in interrupted educational performance, poor learning process, weakened ability, and undeveloped talents. It not only puts their mental health at risk, but also deprives them of a healthy and prosperous life.^15^ Some students are not assertive enough to demand or ask for help from others. Passiveness disables students when communicating with instructors, counselors, and classmates.^16^ In a research, 60% of the students were suffering from lack of communication assertiveness and embracement and this shortcoming had negatively affected their practical learning and performance of 40% of the students.^17^ On the other hand, students with high level of assertiveness were dealing with lower adaptation problems and less frequently suffered from loneliness. These students also indicated higher educational performance, implying their further educational interactions and seeking for help in learning materials.^18^

To develop a successful communication with patients, families, and colleagues, assertiveness is considered as an important hallmark for being a professional nurse. Furthermore, assertiveness is the essence of nursing activities, enabling them to be more independent and make decisions more appropriately.^5^ Assertiveness is considered as a valuable behavior in nursing since it involves positive results, such as enhancing leadership skills, increasing job satisfaction, achieving real independence, professional accomplishment, power and determination, avoiding negligence and overlook during the care giving, decreased job stress, and increased efficiency during the changes in condition. The ability of giving proper assertive response to the critical or potentially risky situations is a vital and life-saving skill. Through an assertive behavior, it is possible to develop appropriate communications and support patients’ rights without harming the professional relationships.^11^

In one study, researchers studied the relationship between depression and assertiveness for subjects and found that there is an inverse statistically significant relationship between them.^19^ The review of literature performed in this study indicated that similar research has been conducted in other communities and target groups and no study was found about the relationship between assertiveness and depression in nursing students. Since nursing students are highly exposed to depression^20^ and assertiveness is among the treatable interpersonal communication aspects,^11^ if a significant relationship is found between these factors, enhancing the level of assertiveness could probably reduce depression in this high risk group. We aimed to determine depression and assertiveness levels and relationship between them in nursing students of Tehran University of Medical Sciences during Autumn and Winter of 2012.

## Materials And Methods

This cross-sectional correlational study was conducted in Tehran University of Medical Sciences in 2012. The study population consisted of bachelor nursing students before entering the clinical field. The inclusion criterion was lack of any psychological problems. Since we aimed to evaluate the relationship between assertiveness and depression as well as investigate assertiveness and depression levels in the students, the sample size was calculated based on a pilot study of 30 nursing students. Forty-four percent of them had high level of assertiveness and 60% had no depression. Thus, the sample size was estimated to be 250 using above result and α=0.05 and 1-β=0.80. For selecting the samples, convenience sampling method was performed. Having no previous information about classes, the researcher selected the students from the courses held while the researcher was present for sampling at the faculty. All students in the selected classes, who were willing to participate in the research, received questionnaires at the beginning or end of the class.

The data collection form consisted of three parts. Part 1 included demographic information such as age, sex, marital status, number of family members, etc. Parts 2 and 3 were assigned for Gambrill and Richey assertion inventory and Beck’s depression inventory, respectively. 

Gambrill and Richey assertion inventory contains 40 items on areas such as turning down friends’ asking for a favor, starting a gap with a stranger, turning down others’ invitations, admiring others, inviting a friend, and ability of expressing feelings. Each item is rated on a 5-point scale ranging from “I never feel upset” (score=1) to “I feel very upset” (score=5). The overall obtained score is within the range of 40 to 200, where a lower score indicates higher assertiveness, and vice versa. Scores on this inventory are classified as follows: 40-79.9 (high), 80-119.9 (average), 120-159.9 (low), and 160-200 (very low).^11^

We the standardized assertiveness questionnaire was used. The validity and reliability of the inventory has been evaluated in Iran. In one study, researchers studied internal consistency of the questionnaire and reported a Cronbach’s alpha of 0.96. They also reported that McCartan and Hargie documented validity and reliability of Gambrill and Richy assertion inventory as 39%-70% and 87%, respectively.^11^ In the present study, the reliability of the instrument was determined as 0.89, using the α-Cronbach’s coefficient.

Beck’s depression inventory is one of the most common depression measurement instruments published by Beck and colleagues in 1961.^21^ The questionnaire contains 21 multiple choice items (scored from 0 to 3) where each expression measures a symptom. The symptoms of this test are classified in three 7-item groups including sensational, emotional, motivational, cognitive, physical, and vegetative symptoms.^22^ Scores 0-13 indicate lack of depression, 14-19 slight depression, 20-28 average depression, and 29-63 severe depression. The questionnaire is translated into Persian and its validity and reliability has been tested. In one study, quoting Green et al, α-Cronbach’s coefficient was reported as 0.92 for this questionnaire.^21^ In our study, the reliability of the instrument using α-Cronbach and Intraclass Correlation Coefficient was reported 0.897 and 0.89, respectively; attesting the reliability of the instrument.

This was approved by the Ethics Committee of Tehran University of Medical Sciences, Tehran, Iran. All ethical considerations for entering the research area were obeyed. After being informed of the research objectives, the participants willingly participated in the research with their informed consent. Moreover, to respect privacy of the participants, the questionnaires were filled out anonymously. Submitting the completed questionnaires to the research indicated their consent for participation in the present research. 

Data were analyzed using SPSS software, version 16. Descriptive and inferential statistics such as Pearson correlation test (to determine relationship between assertiveness and depression), analysis of variance (ANOVA), and independent samples *t* test (to determine the relationship between the demographic variables and main variables of the research) were used. P<0.05 was considered as significant.

## Results

Among the selected students, 248 (92%) (162 freshmen and 86 sophomores) completed the questionnaires and returned it to the researcher. Most students were mostly 18-20 years old, female, unemployed, had 4 to 5 household members, were the first or second child, and moderately interested in their field of study (table 1).

**Table 1:** Comparison of depression and assertiveness scores based on demographic characteristics

**Demographics**		**Frequency**	**%**	**Depression** **mean±SD**	**Assertiveness** **mean±SD**	**Test results** **(Relation with depression)**	**Test results** **(Relation with assertiveness)**
Age	18-20	163	66.8	13.0307± 9.46496	84.2883± 1.9361	F=0.968 df=2,241 P=0.381	F=0.093 df=2,241 P=0.911
	21-24	73	29.9	12.7397± 8.93965	83.2466± 1.94027		
	25≤	8	3.3	8.3750± 6.92691	85.3750± 2.12867		
Sex	Female	163	65.7	12.7669± 9.28664	81.8834± 19.54937	t=-0.93 df=246 P=0.926	t=-2.125 df=246 P=0.035
	Male	85	34.3	12.8824± 9.20328	87.3412± 18.48587		
Employment	Yes	43	17.4	12.8837± 10.64404	83.2791± 20.01586	t=0.058 df=245 P=0.954	t=-0.207 df=245 P=0.836
	No	204	82.6	12.7941± 8.96829	83.9510± 19.22738		
Number of family members	1-3	36	14.9	12.2500± 9.07233	81.7500± 16.63623	F=0.212 df=2,239 P=0.809	F=0.994 df=2,239 P=0.371
	4-5	164	67.8	12.9146± 9.22746	84.9146± 19.80593		
	≤6	42	17.4	12.0000± 8.49103	80.7381± 19.85811		
Birth order	1-2	151	61.1	12.3642± 9.21700	83.9338± 19.96653	F=0.349 df=2,244 P=0.706	F=0.375 df=2,244 P=0.688
	3-4	65	26.3	12.9077± 8.30986	82.1692± 16.95819		
	5≤	31	12.6	13.8065± 10.01805	85.6774± 21.23737		
Interest to field	High	100	40.7	11.0300± 9.18327	80.8100± 18.32517	F=6.828 df=2,243 P=0.001	F=2.168 df=2,243 P=0.117
	Average	123	50	12.8618± 7.52300	84.9756± 19.22450		
	Low	23	9.3	18.5217± 12.83769	88.6957± 23.15842		

As shown in table 2, 55.6% of the students had average to low levels of assertiveness and 38.7% had mild to severe depression. The results showed a significant statistical relationship between depression and assertiveness (r=-0.314; P<0.001). As the correlation coefficient (r) shows, the relationship between these parameters is inverse; increasing assertiveness leads to decreased depression, and vice versa.

**Table 2:** Frequency distribution of depression and assertiveness levels and their relationship

**Depression** **Assertiveness**		**No depression** **(0-13)**	**Mild** **(14-19)**	**Average** **(20-28)**	**Severe** **(29-63)**	**Total**	**mean±SD**	**Test results**
High (40-79.9)	N	85	12	7	6	110	12.8065± 9.23966	Pearson Correlation r=-0.314 P<0.001 Significant
	% within assertiveness	77.3%	10.9%	6.4%	5.5%	100.0%		
	% within depression	55.9%	23.5%	25.9%	33.3%	44.4%		
Average (80-119.9)	N	64	36	19	11	130		
	% within assertiveness	49.2%	27.7%	14.6%	8.5%	100.0%		
	% within depression	42.1%	70.6%	70.4%	61.1%	52.4%		
Low (120-159.9)	N	3	3	1	1	8		
	% within assertiveness	37.5%	37.5%	12.5%	12.5%	100.0%		
	% within depression	2.0%	5.9%	3.7%	5.6%	3.2%		
Total	N	152	51	27	18	248		
	% within assertiveness	61.3%	20.6%	10.9%	7.3%	100.0%		
	% within depression	100.0%	100.0%	100.0%	100.0%	100.0%		
mean±SD	83.754±19.32903							

The results showed that none of the parameters including age, employment, number of household members, and birth order was significantly correlated with assertiveness and depression. There was a significant relationship between depression and interest in the selected field of study (P=0.001) and between assertiveness and sex (P=0.035). 

## Discussion

In this research, Most (55.6%) students indicated average and low levels of assertiveness. In a research conducted on 173 nursing and 77 midwifery students, 59.7% of the nursing students indicated average assertiveness while 11.7% of them exhibited low assertiveness.^11^ Besides, in a semi-experimental study, researchers studied 115 freshman nursing students and reported an average assertiveness level for 65.1% of them.^23^

The results of the present research revealed that 38.7% of the nursing students suffered from mild to severe depression. In one study, similar results were reported: 36.8% of the student had mild to severe depression.^9^ Also, in another study, researchers studied suicide and depression symptoms in nursing students and reported a 44% mild to severe depression levels.^24^ Other researchers reported that 30.8%, 17.7%, and 6.3% of nursing students suffer from mild, moderate, and severe depression, respectively.^8^ However, their study is different from ours  in term of the number of their samples (130 subjects). Besides, they performed their research on nursing students of a military medical sciences university, where the existing stress increased the feeling of insecurity, decreased the performance level, and created depression in students.^8^ These factors might explain the higher depression level in these students as compared with our sample of medical students.

The results of the present study also showed that there is an inverse correlation between assertiveness and depression in nursing students, i.e. the more assertive students are, the less depressed they will be, and vice versa. This finding is in agreement with the results of some studies. In a study, researchers found an inverse correlation between depression and assertiveness in both non‌-blinded and blinded individuals.^25^ Besides, other researchers studied 100 participants with an age range of 18 to 50 years and economic status of poor to average, reporting a strong inverse relationship between depression and assertiveness.^10^ Moreover, in another study the relationship between assertiveness and depression in the residents of the elderly care centers was studied and a negative correlation was reported between them.^19^

According to our results, there was a significant relationship between depression and interest in the selected field of study, as 72% of the students interested in their field of study did not exhibit depression symptoms. In this regard, in one study, researchers reported a significant relationship between depression and interest in the selected field of study. Satisfaction with the selected field of study serves as a strong driving force toward motivation and reduction of the depression prevalence. For any activity, positive feelings about the future and its fruitful end play a key role in dynamicity and prevention of failure and humiliation.^9^ In addition, there was a significant relationship between assertiveness level and the subjects’ sex so that male students were more assertive than female ones. This result is consistent with those of another study.^26^ On the other hand, some others did not report a statistically significant relationship between assertiveness and sex.^27^^,^^28^ It is believed that some of the differences in assertiveness between men and women is attributed to the relationship between assertiveness and socio-cultural factors. Sometimes sex differences are even found in subcultures. Roles and expectations imposed on individuals by culture and parents’ attitudes can lead to the formation of conflicting findings for the relationship between assertiveness and sex.^26^ No significant relationship was observed between depression, age, and sex, consistent with the results found by other researchers.^29^ In another study, a significant relationship was reported between depression, age, and sex;^30^ however, their work was different from the present one in terms of subjects, number of participants, and data sampling process (determining the depression level by interviews).

## Conclusion

A significant inverse relationship was found between assertiveness and depression in nursing students. Therefore, as assertiveness is among the treatable interpersonal communication aspects, probably enhancing the level of assertiveness can reduce depression in this high risk group. Since the studied students were entirely from one department, we suggest future research to study the relationship between assertiveness and depression in other faculties and compare the results with those of other faculties. To generalize this relationship, also a study is suggested to be conducted using random sampling throughout the country. This was a cross-sectional study, and therefore the relationship between depression and assertiveness was examined. Thus, impact of these variables on each other is not clear. We suggest a cohort study design that can determine the effect of variables on each other completely.
